# Prebiotics Plus Probiotics May Favorably Impact on Gut Permeability, Endocannabinoid Receptors, and Inflammatory Biomarkers in Patients with Coronary Artery Diseases: A Clinical Trial

**DOI:** 10.1002/fsn3.3835

**Published:** 2023-11-22

**Authors:** Min Liu, Arash Tandorost, Jalall Moludi, Priyankar Dey

**Affiliations:** ^1^ Department of General Medicine The First Hospital of Shanxi Medical University Taiyuan Shanxi China; ^2^ Research Center for Environmental Determinants of Health (RCEDH) Kermanshah University of Medical Sciences Kermanshah Iran; ^3^ Department of Biotechnology The Institute of Engineering & Technology Patiala India

**Keywords:** coronary artery diseases, endocannabinoid system, gut microbiota, metabolic endotoxemia, prebiotics, probiotics

## Abstract

While gut‐to‐systemic translocation of pyrogenic endotoxin due to a leaky gut elicits systemic inflammation, at the intestine, the endocannabinoid system (eCB) also plays a major role in modulating the impact of gut dysbiosis on the host system. Therefore, we hypothesized that coadministration of prebiotic inulin with probiotics would improve the eCB system, gut microbial composition, and inflammatory parameters associated with coronary artery diseases (CAD). We designed a randomized, double‐blind trial with 92 CAD patients. Patients were randomly allocated to receive inulin (15 mg/day), LGG capsules 1.9 × 10^9^ colony‐forming unit (CFU) or inulin plus probiotic (synbiotics) supplements, for a duration of 60 days. We assessed gut microbiota composition, expression of cannabinoid receptors (i.e., CB1 and CB2), serum levels of interleukin‐6 (IL‐6), toll‐like receptor 4 (TLR‐4), lipopolysaccharides (LPS), total antioxidant capacity (TAC), and malondialdehyde (MDA) before and after the supplementation. Probiotic‐inulin cosupplementation significantly decreased IL6, LPS, and TLR‐4 and increased serum TAC concentrations compared with the placebo. While CB1 receptor expression had no difference, significant differences were observed for the CB2 receptor expression among the four treatments. CB2 receptor mRNA expression significantly (*p* < .05) correlated with serum levels of LPS (*r* = −.10) and F/B ratio (*r* = −.407, *p* = .047). Our data collectively provide preliminary evidence that gut microbiota determines gut permeability through the LPS–eCB system. We also have found that synbiotics improved the eCB receptors, and inflammatory biomarkers more than either of the two supplementations given alone.

## Graphical abstract

- Prebiotic and probiotic administration improved gut permeability through interactions with the eCB system.
- Inulin plus probiotics improved the eCB receptors and inflammatory biomarkers more than either of the two supplementations alone.
- Probiotic plus inulin supplementation improved Firmicutes/Bacteroidetes (F/B) ratio in patients with CAD

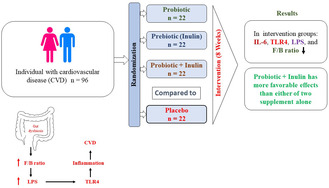

## 1 INTRODUCTION

The link between chronic systemic inflammation and the development of coronary artery disease (CAD) is well established with several clinical and preclinical data supporting a causal relationship (Carding et al., 2015; Eshghinia & Mohammadzadeh, 2013). Indeed, patients with chronic metabolic conditions, such as obesity, fatty liver, and type II diabetes who are at risk of developing CAD, demonstrate increased levels of proinflammatory markers in the circulation (Sekirov et al., 2010). In line, the gut microbiome has emerged as a key regulator of CAD risk as several preclinical and clinical studies indicate alterations in the gut microbial population, diversity, and metabolic functions being associated with CAD (Amar et al., 2013). Although major evidence suggests that the microbiome–CAD association is largely correlative, these data collectively suggest increased gut permeability, chronic ‘low‐grade’ inflammation, redox imbalance, and increased microbial translocation in CAD patients (Boulangé et al., 2016; Lau et al., 2017). Indeed, gut barrier dysfunction facilitates gut‐to‐systemic translocation of pyogenic endotoxin (e.g., LPS), which triggers TLR4/NFκB‐dependent inflammation at the systemic level, which is a major risk factor for CAD development (Moludi et al., 2020).

While the effects of altered gut microbial population and diversity, low‐grade inflammation, and CAD risk have been proposed (Boulangé et al., 2016; Sekirov et al., 2010; Tang et al., 2015), how the gut microbiome‐centric processes induce a leaky gut remains mostly unexplored. Growing evidence supports that the role of the endocannabinoid system (eCB) at the intestinal mucosal may be related to the dysbiosis‐associated low‐grade inflammation (Cani, 2012; Cani et al., 2016). Indeed, the eCB system associated with endogenous lipids can activate specific G‐protein‐coupled receptors (GPCRs) are thought to have cannabinoid receptors 1 and 2 (CB1 and CB2) (Zou & Kumar, 2018), thereby affecting numerous biological processes, such as the regulation of energy homeostasis, mucosal inflammation, and gut barrier function (DiPatrizio, 2016; Witkamp, 2016). In line, others have demonstrated the association of altered expression of intestinal eCB receptors with CAD and type 2 diabetes (de Azua & Lutz, 2019; Moludi et al., 2018; Piscitelli & Silvestri, 2019). Additionally, LPS is identified to stimulate eCB synthesis, while the gut microbiome is suggested as a key contributing factor in this complex regulation (De Laurentiis et al., 2010). While the eCB system is now being targeted in numerous pathological conditions such as CAD, obesity, and diabetes mellitus, certain potential drug candidates also show adverse effects. Thus, there is an urgent need for alternative strategies to limit the risk of CAD by targeting the eCB system in the intestine (Leite et al., 2009).

Probiotics are live microorganisms, which confer health benefits on the host, and positively affect the health of the host by restoring the composition of the gut microbiome (Oelschlaeger, 2010). Several probiotic formulations have shown promising antiobesity, anti‐inflammatory, and antioxidative effects (Cristofori et al., 2021). Functional probiotics mitigate metabolic endotoxemia by limiting mucosal inflammation and gut barrier function (König et al., 2016). Prior data suggest that oral administration of probiotic *Lactobacillus acidophilus* can favorably modulate CB1 receptor mRNA expression (Dothel et al., 2019). Additionally, the levels of tight junction proteins (Occludin and Zonulin) (Chen et al., 2019) were upregulated in a CB1‐dependent mechanism due to probiotic treatment (Cani et al., 2016). These changes in CB1 expression remain related to the amount of bacterial translocation (Chen et al., 2019), whereas CB2 receptor activation may improve glucose tolerance and reduce gut permeability in preclinical models (Zhang et al., 2016).

Other strategies to reduce the risk of CAD included the use of prebiotics including short‐chain carbohydrates such as oligosaccharides and inulin, which can act as substrates for the endogenous colonic symbionts such as *Bifidobacterium* and *Lactobacillus* (Zendeboodi et al., 2020). Prebiotic administration leads to increased production of short‐chain fatty acids (SCFAs: acetate, propionate, and butyrate) which are proposed to improve gut barrier function and have systemic anti‐inflammatory effects (Adhikari & Kim, 2017).

To date, there have been very limited controlled interventions focusing on the metabolic health beneficial effects of coadministration of prebiotic inulin with probiotics in patients with CAD. Therefore, we hypothesized that coadministration of prebiotic inulin with probiotic *Lactobacillus rhamnosus* would improve the gut microbiota composition, gut barrier function, and metabolic endotoxemia in patients with CAD.

## 2 MATERIALS AND METHODS

### 2.1 Subjects

In this trial, 116 eligible patients referring to Imam Ali Cardiovascular Hospital in Kermanshah University of Medical Sciences, Kermanshah, Iran were chosen for the intervention. Criteria for eligibility were as follows: (1) agreed to participate in the study; (2) patients with CAD; (3) age range of 35–55 years. The subjects with chronic renal failure; hemodialysis; patients receiving immunosuppressive, corticosteroid, and anti‐inflammatory drugs; and a history of supplementation with antioxidants or pre/probiotics or vitamins during the prior 8 weeks were excluded in this trial. The consent form was signed by each participant before the start of the study. The study protocol was in accordance with the Helsinki Declaration of the World Medical Association (2000) and was confirmed by local ethics committee of Kermanshah University of Medical Sciences (IR.KUMS.REC.1398.1065) and was recorded in the Iranian Registry of Clinical Trials (https://fa.irct.ir/user/trial/45357/view) (IRCT20180712040438N4).

### 2.2 Sample size

We calculated the sample size according to a mean reduction in LPS levels, a 5% plausible for loss of follow‐up, and a power of 80% (Moludi et al., 2021). The calculated sample size was 24 subjects for each of the four groups; overall, 96 participants were included. Due to incomplete data, the final analysis included only 92 participants, with 23 subjects in each group.

The main outcomes were the alterations from baseline in toll‐like receptor 4 (TLR‐4) and lipopolysaccharides (LPS). The secondary endpoints were the changes in serum levels of interleukin‐6 (IL‐6), total antioxidant capacity (TAC), and malondialdehyde (MDA).

### 2.3 Randomization and intervention

The study was planned as a prospective double‐blind, four‐arm parallel randomized controlled trial using restricted randomization by the Random Allocation Software. The random sequence was kept unaware and administered by an independent third investigator of the study and clinical process until all the outcome data collection was completed.

**FIGURE 1:**
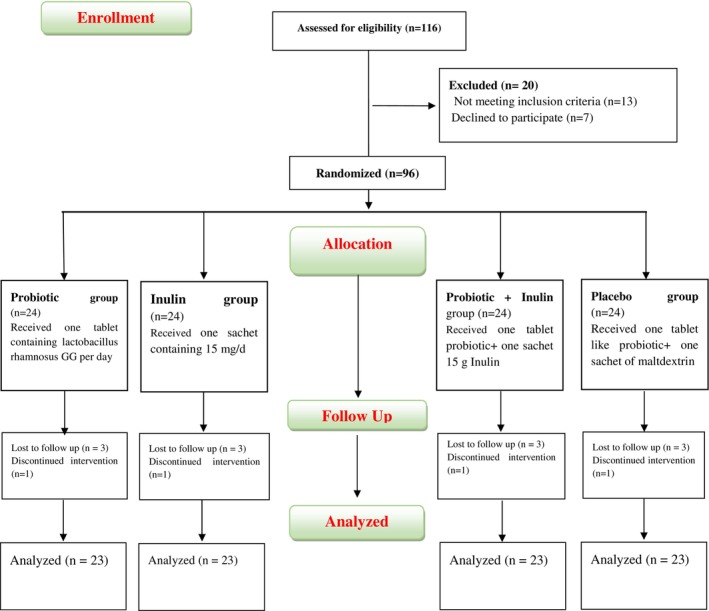
Flow chart of participants screened, enrolled, allocated, and included in the analysis. Ninety‐six participants completed the intervention and were included in the analysis.

The participants were allocated to four groups including; (1) Supplemented with inulin (one sachet containing 15 mg/day); (2) *L. rhamnosus* contained 1.9 × 10^9^ colony‐forming unit (CFU); (3) inulin plus probiotic (synbiotics) groups; (4) Placebo (inulin like sachet and rhamnosus like capsule filled with malt dextrin) as the control group for two consecutive months. Supplements and placebo manufactured by Tak Gene Company (Tehran, Iran) (Figure 1, Study Flowchart).

The compliance was monitored every week by phone calls. The contributors were permitted to stop the trial if they were unwilling to complete or experience any adverse effect during the supplementation.

### 2.4 Dietary intakes

Dietary consumption was measured using 24 recalls at weeks 0 and 8 of intervention. We used Nutritionist IV software which is synced for Iranian diets to acquire nutrient intake information of participants.

### 2.5 Physical activity assessment

The physical activity was assessed to monitor patients’ usual physical activity levels during the study via International Physical Activity Questionnaire (IPAQ).

### 2.6 Assessment of anthropometric and body composition

Body weight without shoes was measured via a scale with an accuracy of 0.250 kg (Seca, Hamburg, Germany). Height was measured without shoes by a measuring tape with an accuracy of 0.5 cm. BMI was calculated by dividing weight (Kg) by height^2^ (m^2^).

### 2.7 Biochemical parameters

After a 10–12 h of fasting, blood was collected and serum was separated from whole blood via centrifugation at 2500g for 10 min. Serum LPS and inflammatory markers including toll‐like receptor 4 (TLR‐4), and interleukin 6 (IL‐6) were measured using enzyme‐linked immunosorbent assay (ELISA) kits (Shanghai, China). Serum high sensitivity C‐reactive protein (hs‐CRP) level was measured by immunoturbidimetry. The serum concentration of malondialdehyde (MDA) using the method of thiobarbituric acid which measures MDA‐reactive products and the total antioxidant capacity (TAC) was assessed by available kits (Glory Science Co). Sera levels of total cholesterol (TC), high‐density lipoprotein cholesterol (HDL‐C), and triglyceride (TG) were assessed by enzymatic kits (Pars Azmun, Iran). Friedewald formula was used to compute low‐density lipoprotein cholesterol (LDL‐C) levels. The fasting blood sugar (FBS) level was measured via the glucose oxidase technique using a commercial kit (Pars Azmun, Iran).

### 2.8 Blood pressure

Systolic blood pressure (SBP) and diastolic blood pressure (DBP) were assessed by an automatic oscillometric device (Omron Healthcare Co., Ltd) after 5 min of rest.

### 2.9 RNA preparation and quantitative Real‐Time PCR

**FIGURE 2:**
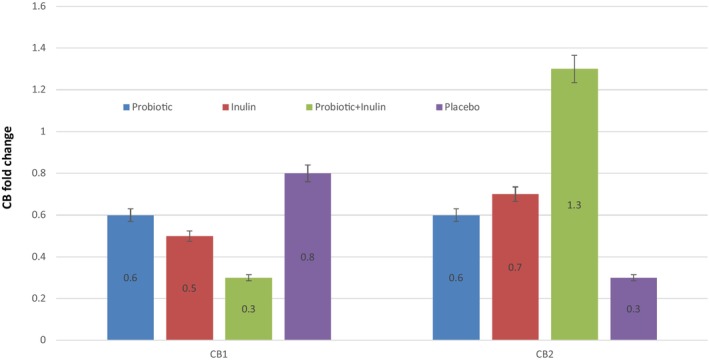
Cannabinoid (i.e., CB1 and CB2) receptor expression, the abundance of RNA was calculated as Relative Expression = 2^−ΔΔC^, shown as fold change in Figure 2.

Total RNA was extracted from PBMC cells isolated from whole blood using Macherey‐Nagel, Germany. The total RNA concentration was measured by a NanoDrop® ND1000 (Thermo Fisher Scientific Inc., Waltham, MA, USA) at 260 nm and 280 nm (A260/A280 ratio). Reverse transcription of total RNA (500 ng per sample) was done by reverse transcriptase (Bio‐Rad, Hercules, CA, USA), as reported by the manufacturer's procedure. qPCR reactions were accomplished via TaqMan Universal PCR Master Mix (Applied Biosystems, Foster, CA, USA), consistent with the manufacturer's procedure. “Primer pairs used in the study were: ‐Actin‐F: 5 ‐CTGGAAC GGTGAAGGTGACA‐3; ‐Actin‐R: 5 ‐AAGGGACTT CCTG TAACAATGCA‐3; CB1‐F: 5‐CAGAAGAGCATCATCCACACGTCTG‐3; CB1‐R: 5‐ATGCTGTTATCCAGAGG CTGCG CAG TGC‐3; CB2‐F: 5 ‐TTTCCCACTGATCCCCAATG‐3; CB2‐R: 5 ‐AGTTGATGAGGCACAGCATG‐3 (Rotter et al., 2013)”. Reactions were done on a Real‐Time PCR Cycler IQ (Bio‐Rad, Hercules, CA, USA) with version 3.0 of the software. Cycle threshold values (Ct) were calculated automatically. Expression of the β‐actin transcript (housekeeping gene) was measured to control for variation in cDNA amounts. The abundance of RNA was calculated as Relative Expression = 2^−ΔΔC^ (Smith et al., 2004), shown as fold change in Figure 2.

### 2.10 Real‐Time PCR for assessment of gut microbiota

Fecal samples were collected in a sterile bottle. We measured the quantity of Firmicutes and Bacteroidetes (F/B) ratio. Real‐time PCR (SYBR green) was used for quantification (the Rotor gene 3000, Corbett Life Science, Mortlake, Australia). Each RT‐PCR reaction mixture contained 10 mM of each primer of Firmicutes and Bacteroidetes, 5‐μL SYBR Green Master mix (Fermentase, Waltham, MA, USA), and 2‐μL DNA template in a 10‐μL final reaction volume. The F/B ratio was calculated using CFU counts (Furet et al., 2009). In healthy subjects, 80% of the recognized fecal microbiota can be categorized into three main phyla: Firmicutes, Bacteroidetes, and Actinobacteria. Generally, the F/B ratios are observed to be of important relevance in human gut microbiota composition (Armougom & Raoult, 2008).

### 2.11 Statistical analysis

All data were analyzed via SPSS software (version 21; SPSS Inc., Chicago, IL) and the outcomes were stated as mean ± SD. For all statistical tests, a *p* value less than .05 was interpreted as statistically significant. The analyses were performed using an intention‐to‐treat (ITT) approach (Wright & Sim, 2003). In order to determine normality, we used the skewness and kurtosis test. Within‐group comparisons (end‐point vs. baseline) were done by paired samples *t*‐test. Comparison of the four groups was done by the analysis of covariance (ANCOVA) followed by the Sidak test after adjusting for the baseline and confounders.

## 3 RESULTS

### 3.1 Baseline data

The flow diagram of the study is exemplified in Figure 1. Based on ITT principles, all patients are included in the analysis. The patients' baseline features are accessible in Table 1. The mean age of total participants was 51.25 (13.22) and no significant difference was observed between groups (*p* = .153). No statistically significant difference was perceived in other baseline parameters including sex, family history of diseases, physical activity, and smoking between the groups (*p* > .05). Adherence to intervention in the current trial was above 75% in all groups.

**TABLE 1:** Baseline characteristics of patients with coronary artery diseases.

Variable	Probiotic group (*n* = 24)	Inulin group (*n* = 24)	Synbiotic group (*n* = 24)	Placebo group (*n* = 24)
Age (years)^b^	51.25 (12.66)	52.18 (12.78)	49.12 (11.22)	51.82 (12.22)
Sex^a^				
Male	15 (62)	12 (50)	15 (62)	16 (66)
Weight (before intervention)^b^	81.27 (12.96)	79.22 (11.42)	78.48 (9.96)	80.46 (10.70)
Family history of CAD^a^	3 (12)	4 (16)	6 (18)	7 (19)
Smoking^a^	5 (20)	4 (16)	7 (28)	3 (12)
Physical activity ^a^				
Low	18 (81)	16 (72)	17 (77)	17 (77)
Moderate	4 (14)	6 (24)	6 (24)	5 (18)
F/B ratio(before intervention)	5.10	5.81	5.07	5.69
F/B ratio (after intervention)	2.87	3.70	1.28	5.21

Abbreviation: F/B, firmicutes to bacteroidetes.

^a^ Values are expressed as frequency (%).

^b^ Values are expressed as mean (SD).

A significant decrease in the F/B ratio was identified in subsequent probiotic plus inulin supplementation as compared to placebo. Accordingly, probiotics + inulin supplementation led to an improvement of the gut microbiota's bacterial genera compared with placebo. There were no significant differences in energy intake and weight among the four groups (Table 1).

### 3.2 Inflammation, oxidative stress, and level of microbial translocation factors

The changes in the serum levels of inflammatory and oxidative stress factors, TLR4, and LPS, within and between the interventions and placebo groups, are presented in Table 2. At the baseline, no significant difference was perceived in TAC, MDA, IL‐6, LPS, and TLR4 between the groups. After 8 weeks' intervention, a significant reduction was found in IL‐6, TAC, and LPS levels in the probiotic (*p* = .007, *p* = .031, *p* = .014, respectively) and probiotic + inulin (*p* = .001, *p* = .003, *p* = .010, *p* = .009, respectively) groups in comparison with baseline. Also, in the probiotic + inulin group, TLR4 level was significantly lower compared to the placebo group (*p* = .033). These results indicate that probiotic + inulin combination could be a feasible approach to attenuating chronic inflammation, endotoxemia, and clinical outcomes in CAD patients.

**TABLE 2:** Baseline value and mean changes of inflammatory and oxidative stress markers, and level of microbial translocation within and between study groups assessed before and after 8 weeks' intervention.

Variable	Probiotic group (*n* = 23)	Inulin group (*n* = 23)	Synbiotic group (*n* = 23)	Placebo group (*n* = 23)	*p*‐value
IL‐6 (ng/dL)					
Before	8.39 (3.85)	7.52 (3.1)	8.37 (3.80)	8.19 (4.12)	.973^a^
After	5.03 (1.07)	6.20 (1.98)	4.18 (1.55)	7.85 (1.67)	.026^b^
GMD, *p* ^c^	−3.39, **.007**	−1.32, .636	−4.39, **.001**	−0.44, .636	
MDA (nmol/mL)					
Before	162.52 (66.85)	166.89 (55.1)	178.29 (82.80)	169.10 (62.66)	.957^a^
After	149.50 (56.1)	141.84 (55.98)	116.78 (42.35)	173.20 (66.67)	.232^b^
GMD, *p* ^c^	−13.02, .707	−25.05, .174	−61.51, .045	−3.90, .887	
TAC (mmol/L)					
Before	113.52 (66.85)	125.98 (55.1)	124.29 (82.80)	123.10 (62.66)	.768^a^
After	172.52 (55.85)	174.89 (57.12)	204.12 (77.88)	116.2 (62.33)	.025^b^
GMD, *p* ^c^	59.00, **.031**	51.11, .086	79.83, **.003**	6.90, .632	
TLR4 (ng/ml)					
Before	14.70 (4.85)	16.16 (3.1)	15.16 (2.98)	13.70 (3.21)	.833^a^
After	9.83 (0.1.00)	12.36 (0.98)	8.50 (0.55)	13.49 (0.67)	.039^b^
GMD, *p* ^c^	−4.70, .086	−3.81, .108	−6.66, **.033**	−0.21, .552	

*Note*: Values are expressed as geometric mean (minimum, maximum) and the p‐values are estimated after log‐transformation.In each raw, mean values with different superscript letters are significantly different (*p* < .05).

Abbreviations: GMD, geometric mean difference; LPS, Lipopolysaccharides; MD, mean difference; MDA, Malondialdehyde; TAC, total antioxidant capacity; TLR4, Toll‐like receptor 4.

^a^ One‐way ANOVA.

^b^ Adjusted for baseline values, age, and sex using the analysis of covariance (ANCOVA) test.

^c^ Paired‐samples *t*‐test.

### 3.3 CB receptor expression

There were no significant group differences in CB1 receptor expression among the four groups (ANOVA, d.f. = 2.51, *F* = 6.31, *p* = 055), while the expression of CB2 receptor was significantly different (ANOVA, d.f. = 2.14, *F* = 4.76, *p* = 003; Figure 2), as confirmed by post hoc pairwise comparison. Moreover, CB1 receptor mRNA expression correlated with the hs‐CRP (*r* = .136, *p* = .044), LPS (*r* = .180, *p* = .037), and F/B ratio (*r* = −.407, *p* = .047). The CB2 receptor mRNA expression inversely correlated with LPS (*r* = −.10, *p* = .040). Lipid profiles, weight, and BMI did not affect the association of these variables (Table 3).

**TABLE 3:** Correlation between CB1 and CB2 receptor mRNA expression with some CAD risk factors and anthropometric indices of patients with coronary artery diseases.

Variables	CB1		CB2	
	*r*	*p* value	*r*	*p* value
Weight	.088	.417	.133	.174
LDL	.141	.148	.110	.120
HDL	.130	.077	.162	.095
Hs‐ CRP	.136	**.044**	.112	**.016**
TAC	.279	.692	.017	.806
Il‐6	.198	.180	.200	.172
BMI	.137	.102	.126	.128
LPS	.180	**.037**	−.10	**.040**
TLR4	.133	.186	.027	.804
F/B	.407	**.047**	.260	.168

*Note*: *p*‐value and *r* is obtained by Pearson correlation.

Abbreviation: F/B, firmicutes to bacteroidetes.

After categorizing the subjects based on at least a 50% increase in CB2 receptor mRNA expression, they were divided into two groups: those with altered CB2 cannabinoid receptor mRNA expression and those with unaltered CB2 receptor mRNA expression. The study results revealed that individuals with altered CB2 receptor mRNA expression had significantly lower cardiovascular risk factors than those in the unaltered group (as shown in Table 4). These findings suggest that an increase in CB2 receptor mRNA expression due to prebiotic or probiotic treatment was linked to a decrease in inflammation, metabolic endotoxemia, and improvement in cardiovascular risk factors. Therefore, the results indicate that the associations between some cardiovascular risk factors were not only related to the modulation of intestinal parameters due to pre/probiotic supplementation but also likely associated with the modulation of the eCB system.

**TABLE 4:** Effect of CB2 receptor mRNA expression on FBS, Lipid profile, blood pressure, and some cardiovascular risk factors of patients with coronary artery diseases.

Variable	Altered CB2 cannabinoid receptor mRNA expression (*n* = 27)	Unaltered CB2 cannabinoid receptor mRNA expression (*n* = 61)	MD (95% CI), *p*‐value^a^
FBS (mg/dL)	97.22 (22.3)	103.97 (18.2)	−6.75 (−12.1 to 23.2), .138
Total cholesterol (mg/dL)	150.14 (49.9)	156.30 (31.9)	−1.13 (−43.1 to 44.2), .957
TG (mg/dL)	133.55 (66.4)	158.66 (53.46)	−25.11 (−33.8 to‐1.7), **.035**
LDL (mg/dL)	83.43 (48.9)	92.56 (26.9)	−9.13 (−46.6 to 34.6), .804
HDL (mg/dL)	44.82 (7.50)	43.84 (5.4)	−0.98 (−5.31 to 7.7), .755
SBP (mmHg)	121.58 (14.7)	125.50 (12.1)	−3.92 (−15.8 to 9.7), .555
DBP(mmHg)	79.40 (8.80)	84.10 (11.70)	−6.50 (−3.17 to 16.1), **.048**
Hs‐CRP (mg/dL)	1.76 (0.63)	3.64 (0.93)	−1.87 (−3.02 to −0.6), **.006**
TAC	121.82 (55.50)	101.84 (52.4)	20.02 (−17.31 to 7.7), .755
Il‐6 (mg/dL)	4.48 (1.7)	9.1 (3.1)	−4.71 (−7.18 to −2.7), **.001**
LPS	13.70 (6.50)	31.17 (6.4)	−17.47 (−22.3 to −6.7), **.001**
TLR4	8.47 (4.7)	15.15 (8.1)	−6.67 (−11.8 to 2.7), **.006**

*Note*: For abbreviations, see Table 1. Values are expressed as mean (SD).

^a^ Independent samples *t*‐test.

## 4 DISCUSSION

The current study, as a first trial, evaluated the effects of coadministration of prebiotic inulin with the probiotics *Lactobacillus rhamnosus* on chronic inflammation, oxidative stress, endotoxemia, and eCB system in patients with CAD.

Our results indicated that 8 weeks' supplementation of probiotic plus inulin improved gut bacterial population as reflected by a significantly reduced ratio of Firmicutes/Bacteroidetes (F/B) in patients with CAD. The gut microbiota, as a highly complicated and diverse ecosystem, affects every aspect of human health and disease (Armougom & Raoult, 2008; Sen et al., 2017). The Firmicutes‐to‐Bacteroidetes ratio, as an overall indication of health status, might be influenced by the quality of diet and environmental factors. Firmicutes are commonly associated with a less favorable metabolic profile and taking into account that the F/B ratio is influenced by diet (Claesson et al., 2012; Sen et al., 2017; Tremaroli & Bäckhed, 2012), a decrease in F/B ratio following probiotic plus inulin supplementation could highlight the beneficial effect of this supplementation in CAD patients regarding improving gut microbiota. It is demonstrated that imbalances in the gut microbiota may be a chief player in the acceleration of inflammatory processes in the cardiovascular system. Inflammatory processes participate in the atherosclerosis manifestations development (Sanchez‐Rodriguez et al., 2020). The main findings of this study would be that the inulin plus rhamnosus combination reduced endotoxemia and inflammatory markers to a greater extent than the inulin or rhamnosus alone. We have found significant reductions in IL‐6, hs‐CRP, TLR4, and LPS levels in the probiotic plus inulin group compared to the placebo group. These outcomes are in accordance with earlier studies' findings that report the immune system modulation and anti‐inflammatory response, increased activity of antioxidative enzymes, and processes that protect cells against oxidative stress damage following administration of synbiotics (a combination of probiotics with prebiotics) (Kazemi et al., 2020; Plaza‐Díaz et al., 2017). A recent systematic review and meta‐analysis of randomized controlled trials reported that synbiotic administration decreased inflammatory factors more effectively than probiotics alone. Moreover, *L. rhamnosus* combinations were highly effective in modulating the immune system (Kazemi et al., 2020). Therefore, modifying the gut microbiota through the combination of prebiotics plus probiotics could be a concern, for reducing bacterial translocation and endotoxemia, which led to decreased activation of the inflammatory cascade.

**FIGURE 3:**
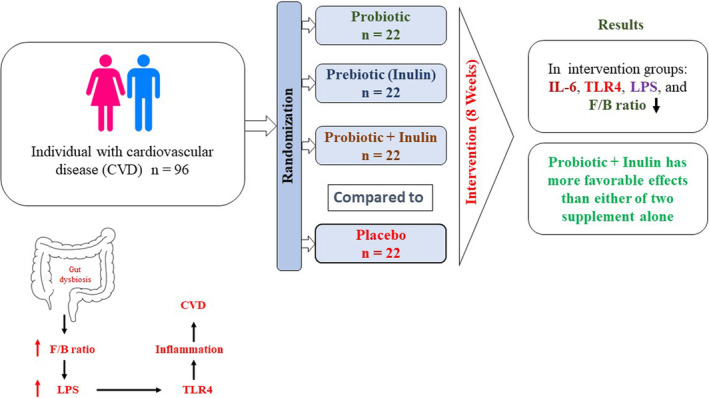
In an 8‐week intervention of individuals with coronary artery diseases (CAD), probiotic and prebiotic (inulin) supplementation improved the eCB receptors, metabolic endotoxemia as indexed by LPS, and inflammatory biomarkers. We also have found that inulin plus probiotics improved the eCB receptors, and inflammatory biomarkers more than either of the two supplementations given alone. Abbreviation: Endocannabinoid (CB2) receptors, lipopolysaccharides (LPS), trimethylamine N‐oxide (TMAO), malondialdehyde (MDA). Graphical abstract is designed with Inkscape software.

Chronic gut inflammation is the main cause of many inflammatory diseases, including CAD, and it has been clearly demonstrated that the eCB systems have a major contribution in modulating this inflammation (Cani et al., 2016; Zhang et al., 2016). The eCB system is an intercellular system with various bioactive lipids, enzymes, and definite kinds of receptors named CB 1 and CB 2 (Rotter et al., 2013). The cells of the cardiovascular system, including infiltrating immune cells, endothelial, vascular smooth muscle cells, and cardiomyocytes, are the expression sites of both CB1 and CB2 receptors (Fulmer & Thewke, 2018). To date, no study has been reported in the literature that indicates the association of eCB system in CAD and its modulation by the administration of the probiotics. In the current study, for the first time, we investigated the eCB system tone (expressions of CB1 and CB2 receptor mRNA), and we found that coadministration of prebiotic inulin with the probiotics *lactobacillus rhamnosus* led to significant upregulation of CB2 receptor expression without significant changes in CB1 receptor expression. Pacher et al. (Pacher & Mechoulam, 2011) demonstrated the raised extents of CB2 receptor expression in the cardiovascular system, under pathophysiological statuses, in particular, inflammatory promotion or tissue damage. This outcome is in line with the findings by Rajesh et al., and Ramirez et al. (Rajesh et al., 2007) that revealed the upregulation of CB2 receptor expression in stimulated human endothelial and smooth muscle cells by proinflammatory stimulants and/or mitogens, which may be indicative of a protective reaction to cell limitation or tissue injury (Ramirez et al., 2012). In this regard, we found that cardiovascular risk factors were significantly lower in those with altered CB2 receptor mRNA expression than those who experienced unaltered CB2 cannabinoid receptor mRNA expression (Table. 4). Accordingly, previous studies showed that the implementation of CB2‐selective ligands enforces the anti‐inflammatory effects on multiple immune cells via mitigating the production of reactive oxygen species and downregulating cytokine release (Correa et al., 2005; Fulmer & Thewke, 2018; Rajesh et al., 2008). Therefore, our findings verified that upregulated expression of CB2 receptor mRNA owing to coadministration of prebiotics with the probiotics is associated with low inflammation, decreased metabolic endotoxemia, and improved some cardiovascular risk factors. Moreover, it is indicated that not only the gut modulation but also modulation of eCB systems due to supplementation with pre/probiotics were involved in the observed improvement of some cardiovascular risk factors. Noteworthy, we observed that the eCB system tone was normalized through the modulation of the gut microbiota via prebiotic plus probiotic supplementation. These outcomes are consistent with the findings of existing evidence, which reported that inflammatory status and its associated metabolic disturbances are controlled through gut microbiota (via LPS), and LPS, as a potent stimulator of the eCB system, can stimulate the eCB system (De Laurentiis et al., 2010). The hyper‐activated intestinal eCB system results in heightened gut permeability, which leads to increases in circulating LPS levels and systemic inflammation. It is suggested that the bidirectional interconnection between the eCB system and the gut microbiota impressed with the resultant modulations due to the combined probiotic and prebiotic intake (He & Shi, 2017). In this regard, another study reported that in vivo and in vitro experiments support the regulatory role of the eCB system on gut barrier function via a CB1‐relevant mechanism. They reported that the CB1 receptor blockade with pharmacological agents improves the gut barrier and reduces metabolic endotoxemia in obese subjects. That was the initial announcement about the regulatory roles of CB1 receptors on gut permeability through interactions with the gut microbiota and the beneficial impacts of the CB1 receptor blockade on protection against obesity and low‐grade inflammation (Muccioli et al., 2010). We found that the expression of CB1 receptor mRNA did not change significantly. According to the study by Cani, it seems that probiotic and prebiotic cosupplementation thoroughly modifies the gut microbiota composition as described, which creates a decreased or normalized eCB system tone in the targeted tissues, thereby counteracting gut permeability and metabolic endotoxemia (Cani, 2012; Cani et al., 2016) (Graphical abstract, Figure 3). However, additional trials are required to clarify the underlying mechanisms that relate to the favorable consequences of coadministration of prebiotic inulin with the probiotics *Lactobacillus rhamnosus* in patients with CAD.

### 4.1 Limitation of study

This study has several limitations that should be considered. First, the period of intervention is moderately short as well. Second, the serum levels of the main endocannabinoids, N‐arachidonoylethanolamine (AEA or anandamide), and 2‐arachidonoylglycerol (2‐AG) were not assessed. In addition, we did not evaluate genetic and imaging biomarkers.

## 5 CONCLUSIONS

Our results support our hypothesis stating that an 8‐week coadministration of prebiotic inulin with the probiotics *L. rhamnosus* (synbiotics) is a better prophylactic strategy than the use of probiotic and prebiotic alone because of the greater decrease in chronic inflammation, oxidative stress, and level of microbial translocation associated with the higher degree of improvement of gut microbiota in patients with CAD. Another promising finding was that the gut barrier function is regulated by the eCB system through CB1‐ and CB2‐associated mechanisms. Also, we observed that gut modulation with pre/probiotics enhances the gut barrier and diminishes metabolic endotoxemia. In addition, this clinical trial for the first time shows that prebiotic plus probiotic administration modified the intestinal eCB system tone and improved gut permeability through interactions with the eCB system. Although many issues remain to be clarified, it is evident that this supplementation could be a promising complement to more conventional as well as nonpharmacological cardiovascular therapies that are commonly used to prevent the onset and progression of CAD.

## AUTHOR CONTRIBUTIONS

**Min Liu:** Software (equal); supervision (equal); validation (equal); writing – original draft (equal); writing – review and editing (equal). **Arash Tandorost:** Data curation (equal); formal analysis (equal); investigation (equal); writing – original draft (equal); writing – review and editing (equal).

## FUNDING INFORMATION

This research was funded by Natural Science Foundation of Shanxi Province+2010011052‐2 and 2022 Teaching Reform and Innovation Project of Higher Education Institutions in Shanxi Province (J20220324). This funding source had no role in the design of this study and will not have any role during its execution, analyses, interpretation of the data, or decision to submit results.

## CONFLICT OF INTEREST STATEMENT

The authors report that they have no conflicts of interest and that no other individuals besides those listed as authors were involved in the preparation of this manuscript. Additionally, the authors disclose that they have no significant relationships with, or financial interest in, any commercial companies pertaining to this article. The lead author further affirms that this manuscript is an honest, accurate, and transparent account of the study being reported.

## ETHICS STATEMENT

Our study was confirmed by local ethics committee of Kermanshah University of Medical Sciences (IR.KUMS.REC.1398.1065) and was recorded in the Iranian Registry of Clinical Trials (https://fa.irct.ir/user/trial/45357/view) (IRCT20180712040438N4).

## Data Availability

All data generated and analyzed during this study are included in the manuscript.
